# Affinity Assays for Cannabinoids Detection: Are They Amenable to On-Site Screening?

**DOI:** 10.3390/bios12080608

**Published:** 2022-08-06

**Authors:** Mihaela Puiu, Camelia Bala

**Affiliations:** 1 R&D Center LaborQ, University of Bucharest, 4-12 Regina Elisabeta Blvd., 030018 Bucharest, Romania; 2 Department of Analytical Chemistry, University of Bucharest, 4-12 Regina Elisabeta Blvd., 030018 Bucharest, Romania

**Keywords:** affinity biosensor, tetrahydrocannabinol, cannabidiol, on-site detection, roadside testing

## Abstract

Roadside testing of illicit drugs such as tetrahydrocannabinol (THC) requires simple, rapid, and cost-effective methods. The need for non-invasive detection tools has led to the development of selective and sensitive platforms, able to detect phyto- and synthetic cannabinoids by means of their main metabolites in breath, saliva, and urine samples. One may estimate the time passed from drug exposure and the frequency of use by corroborating the detection results with pharmacokinetic data. In this review, we report on the current detection methods of cannabinoids in biofluids. Fluorescent, electrochemical, colorimetric, and magnetoresistive biosensors will be briefly overviewed, putting emphasis on the affinity formats amenable to on-site screening, with possible applications in roadside testing and anti-doping control.

## 1. Introduction

*Cannabis sativa* is an annual flowering plant consisting of several botanical variants that produce terpenes, fatty acids, and flavonoids, alongside the major compounds, the cannabinoids. Over time, cannabis has been used for both therapeutic and recreational purposes because cannabinoids are involved in many physiological processes in animals and plants. [1]. Cannabinoids are produced first as carboxylic acids that are further decarboxylated into their more pharmacologically active homologs by exposure to light, heat, or prolonged storage [2]. More than 100 cannabinoids have been identified so far [3]. Among these, Δ^9^-tetrahydrocannabinol (THC) and cannabidiol (CBD), the decarboxylated forms of Δ^9^-tetrahydrocannabinolic acid (THCa) and cannabidiolic acid (CBDa) are the most sought after by consumers, due to their psychoactive and therapeutic effects.

There are three types of cannabinoids:

- Cannabinoids that are produced within the body, (i.e., anandamide or arachidonoyl ethanolamide, AEA, and di-arachidonoyl glycerol, (2-AG) [1,4];
- Phytocannabinoids (cannabinoids that occur naturally in the cannabis plant) such as THC and CBD [5];
- Synthetic cannabinoids that are hallucinogenic chemicals [4,6].

It has been almost three decades since THC has been used for curative purposes. Various cannabis extracts with settled THC/CBD ratios, as well as pharmaceutical-grade herbal cannabis, have been reported in the treatment of several conditions [7]. The pharmacological attributes of THC are related to its high affinity for the type-1 cannabinoid receptor (CB_1_R) [8]. CBD interacts with both receptors of the human endocannabinoid system (ECS), CB_1_R and type-2 cannabinoid receptor (CB_2_R), although with lower affinities, compared to THC [9]. The regulatory functions carried out by ECS in the central nervous system are cognition, appetite control, and analgesia [10]. The other targets of CBD are the orphan G-protein coupled receptor (GPR55), the transient receptor potential channel subfamily V member 1 (TRPV1), and the peroxisome proliferator-activated receptors (PPARs) [11,12]. Targeting the cannabinoid receptors has the potential to treat various health issues and develop biosensing assays for THC.

The metabolic route of THC starts with its hydroxylation to 11-hydroxy-Δ^9^-tetrahydrocannabinol (THC-OH) in the liver (by cytochrome P450), followed by the oxidation of THC-OH to 11-nor-Δ^9^-tetrahydrocannabinol-9-carboxylic acid (carboxy-THC) [13]. Thus, THC exposure and metabolization may be assessed by monitoring carboxy-THC levels in various body fluids [14]. An individual’s impairment state can be evaluated by the quantitation of THC and THC-OH, since both cannabinoids are psychoactive [15]. The most common matrices tested for THC consumption are plasma and urine, but saliva and hair have been exploited more recently, as they are less invasive.

Although CBD is non-psychoactive, it has been extensively used for its validated benefits in the treatment of various health issues such as epilepsy, chronic pain, insomnia, and addiction [4,16]. The CBD formulations have been granted the ‘‘Orphan Drug’’ designation by the US Food and Drug Administration and the European Medicines Agency for use in the treatment of neonatal asphyxia and epilepsy in children [17]. In contrast to THC, CBD is largely excreted intact or in its glucuronide form, as it was reported in recent studies on animals [17]. The degree of CBD exposure can be quantitated by analyzing plasma or urine samples. One advantage of drug testing of saliva over urine is a faster collection process. Another advantage is that a positive result from the saliva test can be confidently assigned to recent drug abuse (within 24 h) and not to a past event, that occurred days to weeks earlier [18,19].

The significant growth of cannabis consumption has heavily impacted the synthetic cannabinoids (SCs) markets. SCs belong to a class of chemicals from the new psychoactive substances (NPS) group, being marketed as natural herbal mixtures under various brand names [6,20]. SCs display mechanisms of action similar to phytocannabinoids, i.e., by binding to the CB_1_ and CB_2_ receptors, thus causing psychoactive effects usually obtained with THC [21]. The abuse of SCs started in the early 2000s, even though their synthesis began in the mid-1960s [22]. The first detected SC in herbal smoking mixtures was JWH-018. [23]. Newly reported SCs ((JWH-122, JWH-210), along with their metabolites, have been detected in biofluidic matrices [24]. The EU Early Warning System on NPSs currently monitors SCs in Europe [24].

Cannabinoids are listed as substances whose use is prohibited in sports competitions along with performance-enhancing substances (PES). Anabolic substances that stimulate muscle growth, and substances that enhance oxygen transport are also included in the PES list [25]; Cannabis abuse should be banned not only during sports competitions but also prior to competitions, considering the time span required for carboxy-THC to clear from urine to a level below the World Anti-Doping Agency’s (WADA) recommended threshold value of 15 ng/mL [26]. The most relevant cannabinoids, along with their chemical structures and functions, are listed in Table 1.

The established detection method of cannabinoids in authorized laboratories is gas chromatography-tandem mass spectrometry GS-MS/MS. However, the GC-MS/MS methods require complex sample derivatization steps and long analysis times [13]. In addition, lateral-flow immunoassay (LFIA) and fluorescence polarization have also been reported [27,28,29,30]. While these methods have provided accurate results, especially in the case of urine samples, there is an impending need to conduct further analytical testing roadside [31].

Overall, roadside testing requires (a) the use of portable, easy-to-handle, and miniaturized equipment (b) non-invasive sample collection with minimum risk of contamination, and (c) adequate analytical methods (fast, sensitive, robust). As in the case of point-of-care (POC) applications, these systems must meet the ASSURED (affordable, sensitive, specific user-friendly, rapid, robust, equipment free, deliverable to end users) criteria. Electrochemical sensing is by far a technique able to match the ASSURED conditions with respect to cannabinoid detection in body fluids. THC detection is based on its electrochemical oxidation, following a reaction path similar to phenol [15]. There are several excellent reviews addressing the electrochemical detection of cannabinoids, based on their simple or mediated oxidation at various modified electrodes [32,33,34,35]. This topic is not covered in the present work, with the focus being put on detection methods based on molecular recognition. The aim of this paper is to assess the feasibility of implementing biosensors in miniaturized and portable platforms for on-site cannabinoid detection in biofluids (blood, urine, saliva, sweat, and breath), especially for roadside testing and anti-doping control.

## 2. Main Matrices for Cannabinoids Screening

The testing manner is heavily dependent on the cannabinoids’ pharmacokinetics. THC appears right away in blood following the first inhalation, with the TCH level reaching a peak within 8 min. Almost 65% of cannabis is eliminated via feces as TCH-OH, while 20% is excreted in urine as carboxy-THC and its glucuronic acid conjugate [27]. The pharmacokinetics of THC and implicitly the half-life depend on the frequency of ingestion [36]. The half-life of THC is about 1–2 days for infrequent users, and around 5–13 days for frequent users [35]. THC can be detected in saliva for up to 34 h after exposure with a 0.5 ng/mL cutoff limit, in plasma for up to 5 h, with a 10 ng/mL limit, and in urine for up to 95 days with a 15 ng/mL cutoff limit [19,37]. Therefore, the sensing methods must be developed according to the actual testing purpose and the complexity of the sample matrix. For example, the early consumption of cannabis may be better assessed by THC levels in saliva or breath, which are the best choices for roadside testing. Rapid screening of carboxy-TCH in serum or urine samples may be required in routine anti-doping control at sports competitions. 

### 2.1. Oral Fluid

Oral fluid (saliva) contains more than 97% water, plus electrolytes, epithelial cells, white blood cells, enzymes (amylase, lipase, peroxidase, and dehydrogenase) immunoglobulins, and glycoproteins (mucin) [38]. Unlike blood, collection of saliva is non-invasive, fast, and can be performed under direct supervision [39,40]. Salivary THC is an indicator of recent drug exposure, while THC-OH is seldom present in saliva [40]. Recent data on impairment levels suggest that a THC concentration of 5 ng/mL in the saliva is equivalent to the legal alcohol limit [41].

### 2.2. Exhaled Breath

Exhaled breath contains a multitude of volatile organic compounds (VOCs) in the vapor phase, and non-volatile compounds impregnated in the suspended solid particles [42]. THC is the most detected cannabinoid in breath, while the levels of carboxy-THC and THC-OH in breath are insignificant. There are few reported devices that collect breath samples and can be used further for THC testing The most common device for sample collection is the SensAbues DrugTrap which utilizes a polymeric filter to capture THC-containing microparticles from exhaled breath [35]. The filter pad is integrated into a plastic collector provided with a mouthpiece to be breathed into by the user [42,43]. The contents of the filter are then dissolved in an organic solvent and made ready for LC-MS/MS analysis [43]. Current breath trapping techniques are operational for roadside testing. Breath has a detection window of 1–12 h and marks recent cannabis exposure [32].

### 2.3. Sweat

Sweat contains 99% water, plus salts, fat, ammonia, urea, and sugars; overall, there are fewer impurities than in other biofluids. Sweat is less prone to degradation [44]. Sweat is collected in a non-invasive manner: a cellulose pad that is applied to the individual for 7–10 days. As the individual sweats, impurities accumulate on the cellulose pad. The patch is removed afterward and spiked with a deuterated internal standard of the compound of interest. A laborious extraction process is carried out to produce a solution for GS-MS/MS analysis; the limits of detection (LODs) have been reported as ng/patch [45]. There are few studies regarding the profile of THC in sweat. A study monitoring THC in the sweat of 11 individuals following interruption of regular consumption, reported a mean THC concentration of 3.85 ng/patch over 7 days [32].

### 2.4. Plasma

Collecting blood samples is an invasive procedure that requires skilled personnel. Electrochemical sensors can detect simultaneously THC and its metabolites in a single blood sample. The ratios of cannabinoid concentrations provide information about the timeline of cannabis ingestion [46]. The THC:THC-OH ratio becomes 2:1 in about 2–3 h after exposure [47]. Plasma concentrations of THC exceeding 2–3 ng/mL have been attributed to recent ingestion [48].

### 2.5. Urine

Urine is the most tested matrix for cannabinoid detection, being reliable, inexpensive, and easy to collect [49]. The factors that influence the time evolution of concentrations and the detectability of THC metabolites in urine include frequency of cannabis usage, the timing of sample collection, body fat content, and degree of urine dilution [50,51]. Carboxy-THC is the relevant analyte in urine testing, since little to no THC or THC-OH can be found in urine [47]. The detection span for carboxy-THC in urine is 33.9 ± 9.3 h [36].

## 3. Trending Methods for Rapid Detection 

In this section, we discuss papers reporting on various methods for rapid detection amenable to on-site testing. The experimental techniques to be described here are electrochemical, fluorescence, and colorimetric. Emphasis will be put on novel strategies for surface modification, and assay principle, as both factors affect sensor efficiency. In the past decade, a new class of sensors (giant magnetoresistive, GMR) gain ground in the bimolecular detection in protein assays using magnetic tags [52].

Modified surfaces can also act as filters that allow only the target analyte to participate in the electrode reaction. In the case of cannabinoids, as in the case of other drugs, the investigation of the mechanism of action within the organism is the basis of biosensor development. A drug’s mechanism of action is based upon its interaction with bio-targets such as antibodies, enzymes, ion channels, receptors, and transporters. Assays that measure the interaction between drugs and their bio-targets are carried out in vitro and may use whole cells or cellular lysates containing isolated bio-targets [53]. Most colorimetric-based detection methods use either competitive enzyme-linked immunosorbent assay (ELISA) or sandwich ELISA formats. However, in the case of cannabinoids’ on-site detection/screening, there is no general affinity concept to be assigned. Most reported biosensors exploit antigen/antibody or ligand/receptor interactions in various formats, either with immobilized or labeled biomolecules, where the label may consist of enzymes, fluorescent, magnetic or conductive nanoparticles. The binding experiments may occur via competition (for antigen/antibody, ligand/receptor, inhibitor/enzyme pairing), modulation (allosteric modulator/enzyme, allosteric modulator/receptor, or in some cases, both ways. In these experiments, the binding of one or more fixed concentrations of a labeled small ligand, (i.e., cannabinoids) is measured at equilibrium in the presence of an incrementing series of concentrations of the non-labeled ligand or allosteric modulator [54].

### 3.1. Electrochemical Detection 

**Scheme 1:**
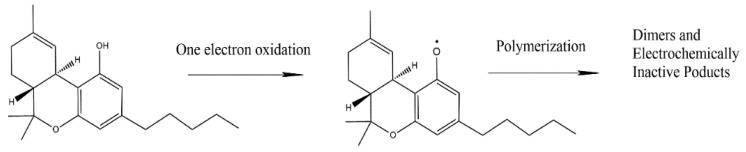
Phenol-like oxidation mechanism of THC as proposed by Balbino et al. [56]. Adapted from [56] (with permission of Elsevier) and from [57] (with permission of Wiley).

Electrochemical techniques are rapid and cost-efficient, widely applied and amenable to the detection of illicit drugs, such as cocaine, marijuana, and ecstasy [55]. The electrochemical techniques related to cannabinoid detection include square wave voltammetry (SWV), differential pulse voltammetry (DPV), and electrochemical impedance spectroscopy (EIS). It is worth mentioning that the direct detection of THC relies on its electrochemical oxidation. As mentioned before, THC is an electroactive compound that undergoes oxidation following a phenol-like mechanism [56]. The reaction route in the basic medium (as proposed by Balbino et al. [57]) involves the deprotonation of the phenolic hydroxyl group, followed by oxidation and formation of a phenoxy radical (Scheme 1). After its formation, the phenoxy radical reacts with another THC molecule or other similar compounds [58]. Despite overwhelming advantages, the electrochemical detection of THC is challenging, because of the hydrophobicity of THC [59].

To overcome the drawback associated with THC’s low solubility in aqueous media or with inherent interferences caused by alcohol solvents, biosensors based on affinity recognition of THC and metabolites by immobilized biomolecules, have been recently spotted. The most relevant papers will be further discussed.

#### 3.1.1. SWV- and DPV-Based Biosensors

In SWV, the staircase potential ramp is modified with square-shaped potential pulses [60]. At each step of the staircase ramp, two opposite potential pulses that are equal in height are imposed. The current sampling is carried out at the end of each potential pulse for decreasing the contribution of the non-faradaic current [61]. DPV is a technique related to SWV. Here, pulses of constant amplitude are superimposed on the potential linear sweep [62]. The current sampling is achieved just before and at the end of the modulation pulse, and the difference is recorded, in order to reduce the contribution of the non-faradaic current [63]. In both SWV and DPV the net current vs. potential pulse displays a symmetrical peak-shaped feature. 

**Figure 1:**
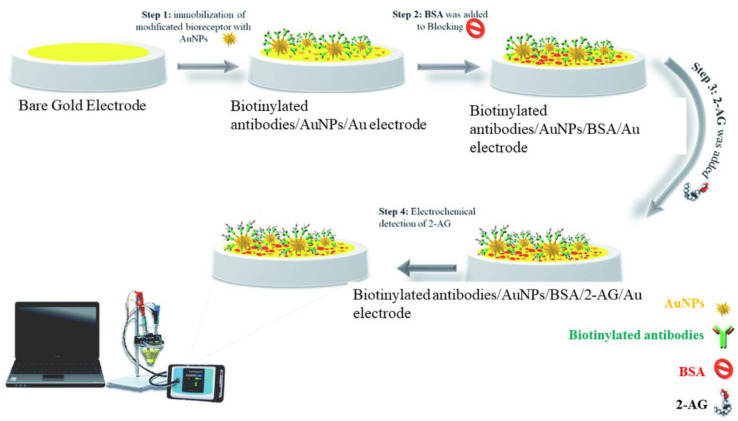
Schematic representation of the immunosensor proposed by Kohansal et al. for the detection of 2-Ag protein conjugate. Reproduced with permission from [64]. This article is licensed under a Creative Commons Attribution 3.0 International License, which permits use, sharing, adaptation, distribution, and reproduction in any medium or format, as long as you give appropriate credit to the original author(s) and the source, provide a link to the Creative Commons license, and indicate if changes were made. The images or other third party material in this article are included in the article’s Creative Commons license unless indicated otherwise in a credit line to the material.

Kohansal et al. have developed an immunosensor for 2-AG in human plasma and rat serum exploiting the conductivity changes at the modified electrode in the presence of the ferricyanide/ferrocyanide system [64]. The interrogation techniques were DPV and SWV. Gold nanostars (GNS) were used to expand the electroactive surface and immobilize Anti-2-Ag antibodies. Firstly, the affinity format exploited the interaction between the surface-immobilized anti-2-Ag antibody and the 2-Ag protein conjugate. The net peak current decreased with the increase in the 2-Ag protein concentration, because of electron transfer blocking between the ferry/ferrocyanide couple and the electrode surface. 2-AG protein was measured with a linear range of 0.48–1 ng/mL and a limit of quantification (LOQ) of 0.48 ng/L. Secondly, 2-Ag was detected in real samples based on the competition between 2-Ag protein and the target 2-Ag for the binding sites of the immobilized anti-2-Ag antibodies. The detection principle was based on the increase in the net peak current with the target (2-AG) concentration. The signal enhancement was accompanied by a slight shift of the redox potential to more negative values. The developed immunosensor showed high sensitivity and specificity in the presence of interferents. Bovine serum albumin (BSA) was used for surface blocking. The preparation of the immunosensor is depicted in Figure 1. This affinity format may be as well applied to the detection of relevant phytocannabinoids or SCs.

#### 3.1.2. EIS-Based Biosensors

According to the relation between the charge transfer and the measured parameters, EIS-based sensors can be categorized into (A) faradaic and (B) non-faradaic (capacitive) [65]:

(A) Faradaic (bio)sensors use electrodes with conductive surfaces; the charge transfer resistance (Rct) is measured in the presence of redox-active species in solution, such as hexacyanoferrate (II)/(III) anions, or hexaammineruthenium (II)/(III) cations [66]. The deposition of non-conductive molecules onto the surface blocks the electron transfer causing an increase in Rct. Conversely, the deposition of conductive molecules or molecules able to catalyze redox reactions determines the decrease in Rct.

(B) Non-Faradaic (bio)sensors are devices containing electrodes with surfaces covered by layer(s) with insulating properties. Here, the double-layer capacitance (Cdl) is the main parameter that characterizes the processes at the electrode interface [67]. The adsorption of molecules onto the surface usually decreases the value of Cdl [68]. Modified electrodes can be used in disposable sensors, to overcome the drawbacks of low signal-to-noise ratios [69].

**Figure 2:**
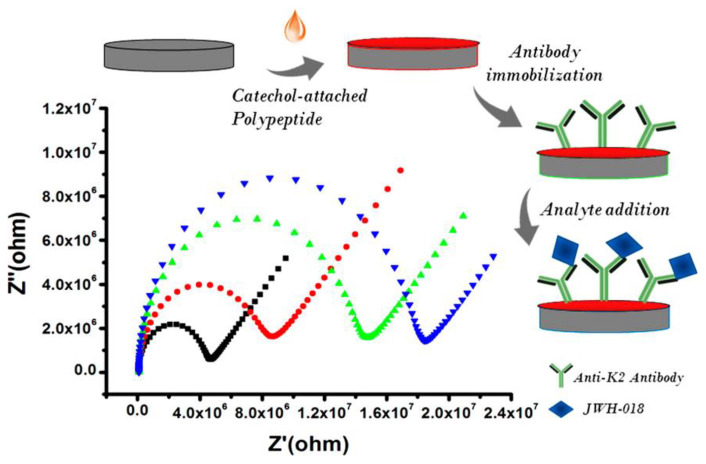
Development of the impedimetric immunosensor for the detection of JWH-018 (*N*-4-hydroxypentyl metabolite. Each step of the sensor development is accompanied by an increase in Rct. Reproduced with permission from [70]. Copyright (2020) American Chemical Society.

Durmus et al. proposed an EIS immunosensor for the detection of JWH-018 (*N*-4-hydroxypentyl metabolite) [70]. First, a functional surface was built up using a catechol-attached polypeptide (CtP). Then, the anti-K2 antibody was incorporated within the polymer via a covalent cross-linker. The modifications, carried out on the glassy carbon electrode surface, are shown in Figure 2, together with the Nyquist plots. The principle of detection lies in the increase in Rct following the direct binding of the target to the functionalized surface. Linearity and the limit of detection for JWH-018 (*N*-4-hydroxypentyl metabolite) were determined as 10–500 ng/mL and 5.892 ng/mL, respectively. The selectivity of the biosensor was evaluated with different interfering molecules (cocaine, codeine, and(methamphetamine). Finally, the immunosensor successfully detected JWH-018 (*N*-4-hydroxypentyl metabolite) in spiked synthetic urine samples. The results showed that the developed platform can be applied to detect other JWH series with high sensitivity and accuracy.

The affinity-based immunosensor developed by Stevenson et al. was able to detect THC in saliva [19]. Non-faradaic EIS was utilized to monitor de binding of a BSA-THC hapten to the capture antibody previously immobilized onto the electrode surface. A linker molecule (dithiobis succinimidyl propionate, DSP) was chemisorbed onto the electrode surface using the gold/thiol chemistry. Then, the capture antibody for the BSA-THC hapten was bound to the surface via the DSP linker. The binding of the hapten to the immobilized antibody was analyzed by non-faradaic changes in the dielectric properties of the electrode/electrolyte interface. The overall measurement of THC took approximately 1 min and provided a LOD of 100 pg/mL.

#### 3.1.3. Chronoamperometric Biosensors

Chronoamperometry (CA) is a potential step method: it measures the current variation vs. time as a response to a variation (step, pulse) in potential. Since the measurements are performed in a transient state, it allows a dramatic reduction of response time compared to amperometry in stirred solution [71]. A sandwich immunoassay based on a double-layer gold nanoparticles-amplification system for THC detection was reported by Lu et al. [72]. Chitosan/gold nanoparticles (GNPs) nanocomposites were deposited onto a glassy carbon electrode (GCE), to absorb horseradish peroxidase (HRP) and thionine (Thi). The anti-THC was trapped between two conductive layers of chitosan/GNP/HRP/thionine. In the presence of H_2_O_2_, the immobilized HRP and thionine were involved in a sequence of consecutive redox reactions that led to the increase in the faradaic current. The binding of the target THC to the immobilized antibody prevented the electron transfer due to steric hindrance. Chronoamperometry was used to determine the THC in phosphate buffer saline (PBS). The results showed that the response current had a good linear correlation with the THC concentration range from 0.01 to 10^3^ ng/mL. The LOD for THC was decreased to 3.3 pg/mL. 

### 3.2. Magnetoresistive Biosensors

**Scheme 2:**
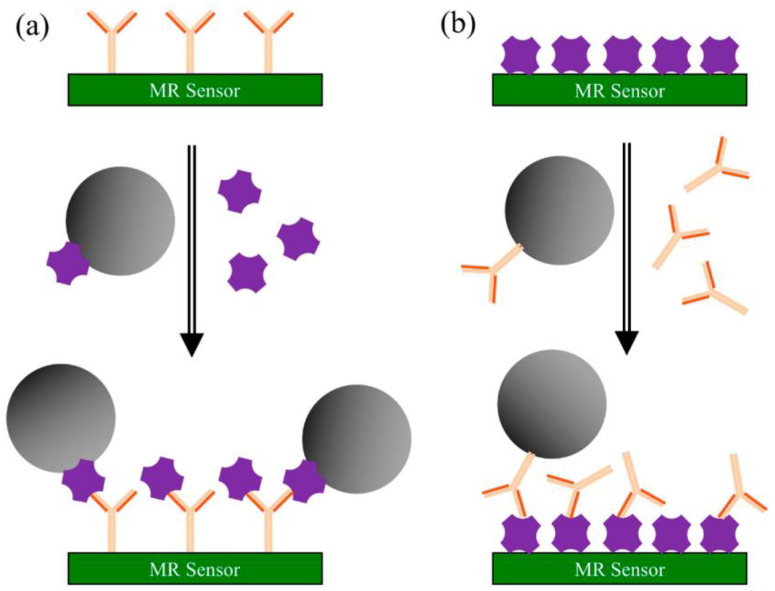
Principle of surface competition assays using MR sensors: (**a**) capture antibodies are pre-coated on the sensor’s surface. A mixture of magnetic nanoparticles (MNPs) labeled antigen and unlabeled antigen from the testing sample is injected onto the surface. Both labeled and sample antigens are competing for the antibody’s binding sites; (**b**) antigens are pre-coated on the sensor’s surface. A mixture of MNPs-labeled antibodies and unlabeled antigens from the sample is injected onto surface. Here, the competition occurs between the immobilized and the free antigen for the MNPs-labeled antibody’s binding sites. In both types of assays, the recorded signal is directly proportional to the number of MNPs bound to the surface. Reproduced with permission from [73]. This article is licensed under a Creative Commons Attribution 4.0 International License, which permits use, sharing, adaptation, distribution, and reproduction in any medium or format, as long as you give appropriate credit to the original author(s) and the source, provide a link to the Creative Commons license, and indicate if changes were made. The images or other third party material in this article are included in the article’s Creative Commons license unless indicated otherwise in a credit line to the material.

Magnetoresistance (MR) represents the change of resistance upon applying an external magnetic field. The capability of converting magnetic signals to electrical signals has led to the successful development of sensing devices based on the MR effect [73]. The GMR effect exists in metallic structures with alternating ferromagnetic and nonmagnetic layers. Following the application of a magnetic field, the magnetization directions of two adjoining ferromagnetic layers become either parallel or antiparallel, depending on the orientation of the external field. GMR biosensing technology has the advantages of low cost, high possibility in portability, high sensitivity, and real-time signal readout [52,74]. The principle of the surface competition assay, adaptable for on-site screening of cannabinoids, is depicted in Scheme 2 [73].

**Figure 3:**
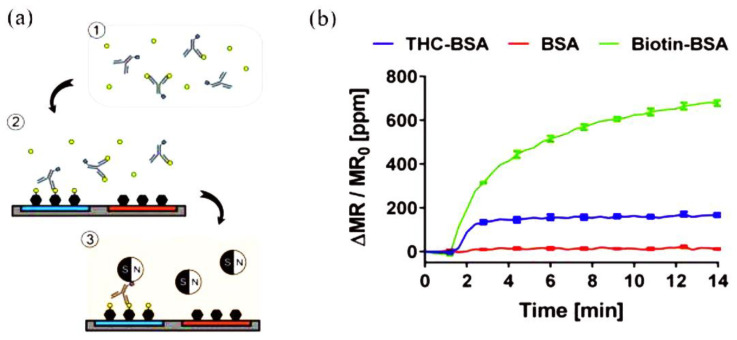
Schematic representation of the GMR biosensing platform for THC: (**a**) 1: biotinylated anti-THC antibodies were incubated with THC-containing samples. 2: the mixture was injected on a chip with immobilized bovine serum albumin (BSA) and THC–BSA bioconjugate, to allow the antibodies to bind. 3: Streptavidin-coated MNPs were added to the chip to communicate the signals. (**b**) Representation of the measured signals of THC-BSA, BSA, and biotinylated BSA (Biotin-BSA) immobilized on different sensors. Adapted with permission from [75]. Copyright (2016) American Chemical Society.

A sensing platform that employs GMR immunosensors integrated with a portable reader system and smartphone to detect THC in saliva using competitive immunoassays was reported by Lee et al. [75]. The competitive assay required only one type of antibody to recognize THC and link the magnetic nanoparticles (MNPs) to THC bound on the electrode surface via biotin−streptavidin interaction. The biotinylated antibodies were added to the sample containing THC to bind to THC in the sample in the preincubation step. Then, the mixture was added to the chip where BSA and THC conjugated with BSA (THC-BSA) were immobilized on different sensors to allow active antibodies to bind to THC-BSA on the sensors. After the washing step, the chip cartridge was inserted into the measurement reader and streptavidin-coated MNPs were added. The stray field from the bound MNPs disturbed the magnetization of the sensors underneath, changing the resistance (Figure 3). The changes in resistance, monitored as GMR signals (ΔMR/MR0), were proportional to the number of bound MNPs and have an inverse relationship with the concentration of THC in the sample due to the nature of competitive assays. THC was detected in the range of 0–50 ng/mL, covering most cutoff values proposed in previous studies. The reported work is amenable to the on-site screening of THC.

### 3.3. Optical Detection

Fluorescence-based detection has grown in popularity, being a swift and on-point detection technique. The popularity of this technique is probably due to its simplicity, high sensitivity, and capability of detecting analytes in small volumes. Moreover, low-cost and simple devices for on-site detection can be designed using a light-emitting-diode (LED) for excitation and photodetector [76]. Fluorescent quantum dots (QDs) techniques have massively improved detection performances. Quantum confinement effects of small nanoscale materials can produce a strong enhancement of fluorescence emission leading to new possibilities over traditional fluorescent organic dye [77]. Lateral flow immunoassay (LFIA) strips coupled with fluorescence analytic devices were used to detect THC from saliva samples.

Plouffe and Murthy [78] reported a fluorescence-based LFIA for THC detection in saliva. Anti-THC antibodies were conjugated to polymeric phycoerythrin-fluorescent particles. The antibodies were used further to capture THC. The positive interaction of THC with the conjugates and the test line produced a fluorescent quantifiable signal. The strategy provided a LOD of 2.57 nM.

LFIA results are often obtained by visualizing the color changes in specific areas, thus providing qualitative information about the presence/absence of the target analyte [79]. Colorimetric detection is an intuitive method for obtaining positive or negative results in LFIA multiplexed detection because the color changes are easy to observe, without the need for special equipment [80,81]. Most of the colorimetric systems involve enzymes with peroxidase activity such as horseradish peroxidase and/or hydrolases such as alkaline phosphatase (ALP) as components of the color development process. Enzymes are preferred in the assays based on color development due to their high specificity toward the target analyte [82]. In all the affinity assays discussed in this review, the recognition elements were antibodies from various sources. Although not so often reported, the cannabinoids’ interaction with the CB_1_ and CB_2_ receptors was exploited to develop activity-based bioassays. A bioassay in which the cannabinoid receptor activation by cannabinoids caused the recruitment of truncated β-arrestin 2 to the cannabinoid receptors, resulting in functional complementation of a split luciferase, allowed readout via bioluminescence [83]. This method seems suitable for a yes/no format around a threshold value (since positive results were obtained for THC levels exceeding 12 ng/mL. It was found recently that CBD and carboxy-THC act as allosteric modulators of the growth hormone secretagogue receptor (GHS-R1a) during its interaction with the endogenous ligand, ghrelin (GHR) [26], and this fact can be further exploited to detect these cannabinoids using a surface competition assay format.

**Scheme 3:**
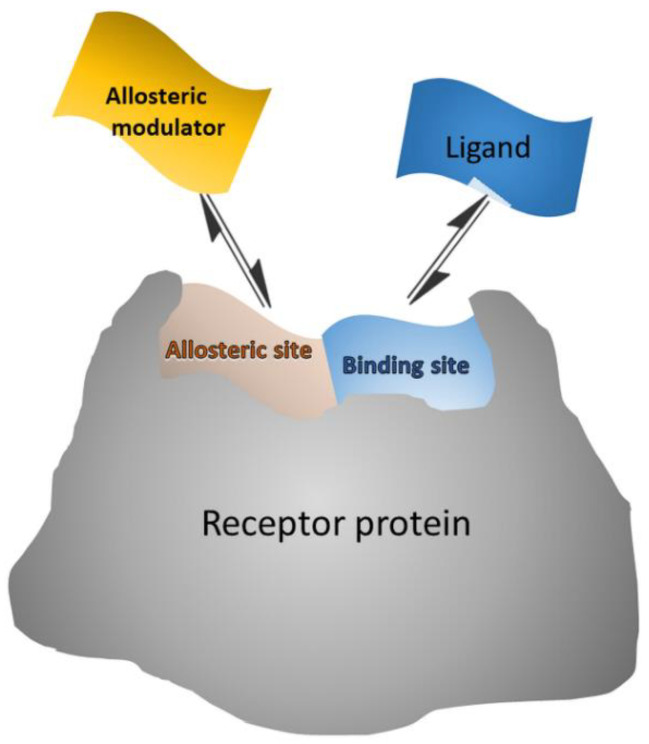
Schematic representation of allosteric modulator/receptor and orthosteric ligand/receptor interactions that can be exploited in surface competition assays, where the surface-bound receptor interacts with both labeled ligand and unlabeled modulator from the sample.

Allosteric modulators are in general small molecules that can bind to a distant site (allosteric site) from the binding (orthosteric) site, causing a functional change of the binding site (Scheme 3). Some allosteric modulators bind both allosteric and orthosteric sites [84]. Thus, the allosteric modulators “push” the conformational equilibrium towards a specific state [85]. Allosteric activators shift the equilibrium towards the active state, where the orthosteric ligand binds with higher affinity, while allosteric inhibitors shift the equilibrium towards the inactive state, where the orthosteric ligand cannot bind [84].

**Figure 4:**
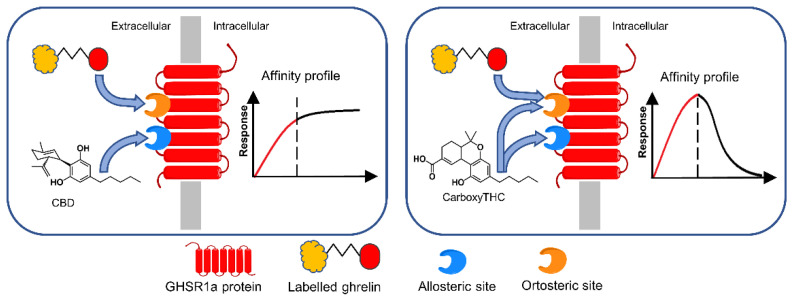
Biosensing principle of CBD and carboxy-THC detection according to their affinity profile in direct competitive assay with HRP-labeled ghrelin. The immobilized recognition element was GHS-R1a. Reproduced from [26] with permission from Elsevier.

Thus, carboxy-THC and CBD were detected via their interaction with the growth hormone secretagogue receptor (GHS-R1a), in competition with the labeled ghrelin GHR) (GHS-R1a’s endogenous ligand) [26] (Figure 4). It was noticed that the two cannabinoids acted as allosteric modulators of GHS-R1a. First, biotinylated anti-GHS-R1a antibodies were coated onto the surface via biotin/streptavidin interaction. Then, the receptor was let to interact with the immobilized antibody, which acted as support for controlled GHS-R1a immobilization. Peroxidase-labeled GHR and cannabinoids competed for the binding sites of GHS-R1a. Finally, after the addition of hydrogen peroxide and chromogenic substrate (tetramethylbenzidine), color development was monitored to build up an affinity profile. The absorbance was directly proportional to labeled GHR bound onto the surface. It was found that CBD strongly enhances the binding of the labeled GHR, probably due to its interaction with the allosteric site of GHS-R1a. The results suggested that CBD binds to the allosteric site only, while carboxy-THC binds to both allosteric and orthosteric sites.

The analysis was performed on spiked urine samples and was able to detect cannabinoids even in the presence of GHR mimetics. The proposed method exploited the linear parts of the competitive binding curves to detect CBD and carboxy-THC. A linear range of 5–30 ng/mL was obtained for both CBD and carboxy-THC. 

A summary of biosensors for cannabinoid detection based on molecular recognition along with their performance features and drawbacks is presented in Table 2.

## 4. Conclusions

THC and its metabolite, carboxy-THC, are important targets for the assessment of cannabis use, with regards to both drugged driving (THC levels), and drug use over time (carboxy-THC levels) [30]. Synthetic cannabinoids such as JWH-018 and JWH-073 have become new targets in both roadside testing and anti-doping control. The recent papers discussed here demonstrate the enormous potential of affinity-based biosensors for cannabinoid detection. Surface competition assay formats involving biofunctionalized transducers and labeled ligands are mandatory for developing portable biosensors for roadside testing. EIS detection does not require the use of labeled biomolecules yet involves several steps that should be performed by trained personnel. DPV and SWV ensure faster and less time-consuming detection provided that redox or enzymatically labeled ligands are used. Modification of the sensor’s surface with various nanocomposites and immobilization of biorecognition counterparts for cannabinoids would result in enhanced specificity, sensitivity, and selectivity. Several biosensors have been able to detect THC in saliva after cannabis exposure The main drawbacks related to these sensing devices are nonspecific adsorption and interference of components from complex sample matrices. Fluorescent and colorimetric LFIA tests provide low-cost, facile, fast, and portable detection and may be the best-suited devices for roadside testing, especially when the samples are collected from saliva GMR biosensors using antigen-pre-coated transducers are also promising alternatives to LFIA tests since they can be implemented in portable and easy-to-use equipment. Nevertheless, any affinity assay uses antibodies or other proteins that require special storage conditions such as being refrigerated at low temperatures. The lifetime of such biomolecules is also limited. Another concern is the fact that the portability of biosensors is still restricted due to issues related to fluid handling, sample preparation, device packaging, integration of electronics for data collection/evaluation, and the need for external power sources. Disposable electrochemical biosensors integrated with microfluidic platforms may deliver the much-sought outcome for on-site screening. Paper-based electrochemical sensors combined with droplet-based microfluidics may balance the requirement of device miniaturization with performance characteristics. The development of stable and long-lasting biosensors with high selectivity in complex matrices and minimum nonspecific interactions is another matter the scientific community must address. Could the techniques discussed here provide reliable commercial devices for real-world on-site screening? Time will tell.

## Figures and Tables

**Table 1:** Chemical structures and main functions of most relevant cannabinoids. Adapted with permission of [32]. Copyright (2020) American Chemical Society.

Cannabinoid	Structure	Function
Δ^9^-tetrahydrocannabinol (THC)	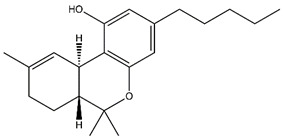	Primary psychoactive component
11-OH-Δ^9^-tetrahydrocannabinol (11-OH-THC)	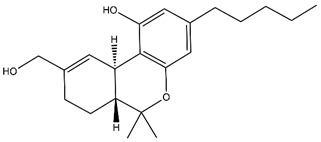	Primary metabolite of THC, psychoactive
11-nor-9-carboxy-Δ^9^-tetrahydrocannabinol (carboxy-THC)	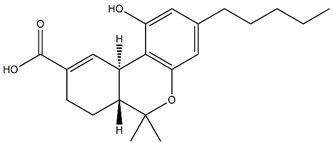	Primary metabolite of 11-OH-THC, non-psychoactive
Cannabidiol (CBD)	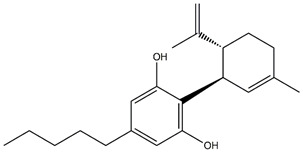	Major component, non-psychoactive, has medicinal use
Cannabinol (CBN)	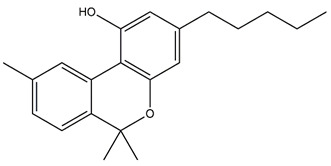	Common product of THC degradation
JWH-073	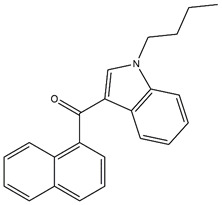	Synthetic psychoactive cannabinoid Mimics the body’s naturally produced endocannabinoid hormones

**Table 2:** Relevant biosensors for cannabinoids’ detection.

Platform	Target	Capturing Element/Assay Format	Detection Technique	Matrix	LOD/Linear Range	Benefits	Drawbacks	Ref.
Paper substrate Whatman grade 1 chromatography	THC	Anti-THC antibodies conjugated to phycoerythrin-fluorescent particles/sandwich	Fluorescence/LFIA	Oral fluid	0.1 ng/mL/Not reported	Rapid detection nonradioactive, high-throughput, low sample volume	Poor stability of the florescent probe, low reproducibility	[78]
Nitrocellulose membrane	JWH-073	Anti-K2 antibodies conjugated to Rhodamine B–loaded polymersome/ sandwich	Colorimetric/LFIA	Saliva	0.53 ng/mL 5.0–1000 ng/mL	High-throughput, low sample volume	Smaller molecules cannot be detected by two antibodies (sandwich assays)	[86]
HBC-2-dimethylamino ethyl methacrylate DMAEMA-modified glassy carbon electrode (GCE)	JWH-073	Anti-K2 antibodies /direct binding	Electrochemical/DPV	Synthetic urine	31.87 ng/mL 25–500 ng/mL	Rapid detection, high specificity	LOD above the cutoff limit for cannabinoids levels	[87]
Chit/AuNP-modified (GCE)	THC	Anti-THC antibodies conjugated to AuNPs/direct binding	Electrochemical/ chronoamperometry	Buffer	3 pg/mL/ 0.01–0.01 × 10^3^ ng/mL	Rapid detection, enhanced sensitivity	Use of labeled reagents, additional signal amplification steps, low stability of nanomaterials	[72]
Au deposited onto polyethylene terephthalate (PET) substrate	THC	Anti-THC antibodies immobilized onto dithiobis(succinimidyl propionate) (DSP) SAM/direct competitive	Electrochemical/ EIS	Human saliva	100 pg/mL/ 100 pg/mL–100 ng/mL	High selectivity, suitable for on-site detection	Extended time range for sensor equilibration, multiple washing steps	[19]
Au electrode	THC	NanoMIP for THC	Electrochemical/ capacitive	Buffer	1.0 × 10^–^^14^ M 1.0 × 10^−^^12^–1.0 × 10^−^^5^ M	High sensitivity suitable for on-site detection	Extended time range for sensor equilibration, multiple washing steps	[88]
AuNP-modified screen-printed carbon array electrodes	THC	Anti-THC antibodies immobilized onto AuNP-modified electrode/direct competitive	Electrochemical/SWV	Spiked urine sample	7 pg/mL/ 10 pg/mL–10 μg/mL	Multiplexing capability, enhanced sensitivity	Risk of cross-reactivity	[89]
ELISA microplates	THC	Plant-produced monoclonal anti-THC antibody/indirect competitive	Colorimetric	Buffer	0.625 μg/mL 0.625–10 μg/mL	High specificity and selectivity	Use of labeled reagents, time-consuming steps	[90]
Functionally activated microplates	Carboxy-THC CBD	Growth hormone secretagogue receptor/direct competitive	Colorimetric	Synthetic urine	5.12 ng/mL/ 5–30 ng/mL 7.63 ng/mL/ 5–30 ng/mL	High sensitivity, low sample volumes	Use of labeled reagents, multiple washing steps	[26]

## Data Availability

Not applicable.
